# New and Rare Carotenoids Isolated from Marine Bacteria and Their Antioxidant Activities

**DOI:** 10.3390/md12031690

**Published:** 2014-03-24

**Authors:** Kazutoshi Shindo, Norihiko Misawa

**Affiliations:** 1 Department of Food and Nutrition, Japan Women’s University, 2-8-1 Mejirodai, Bunkyo-ku, Tokyo 112-8681, Japan; 2 Research Institute for Bioresources and Biotechnology, Ishikawa Prefectural University, 1-308 Suematsu, Nonoichi-shi, Ishikawa 921-8836, Japan; E-Mail: n-misawa@ishikawa-pu.ac.jp

**Keywords:** diapolycopenedioc acid xylosylesters A–C, methyl 5-glucosyl-5,6-dihydro-apo-4,4′-lycopenoate, (3*R*)-saproxanthin, (3*R*,2′*S*)-myxol, antioxidant activity

## Abstract

Marine bacteria have not been examined as extensively as land bacteria. We screened carotenoids from orange or red pigments-producing marine bacteria belonging to rare or novel species. The new acyclic carotenoids with a C_30_ aglycone, diapolycopenedioc acid xylosylesters A–C and methyl 5-glucosyl-5,6-dihydro-apo-4,4′-lycopenoate, were isolated from the novel Gram-negative bacterium *Rubritalea squalenifaciens*, which belongs to phylum Verrucomicrobia, as well as the low-GC Gram-positive bacterium *Planococcus maritimus* strain iso-3 belonging to the class Bacilli, phylum Firmicutes, respectively. The rare monocyclic C_40_ carotenoids, (3*R*)-saproxanthin and (3*R*,2′*S*)-myxol, were isolated from novel species of Gram-negative bacteria belonging to the family Flavobacteriaceae, phylum Bacteroidetes*.* In this review, we report the structures and antioxidant activities of these carotenoids, and consider relationships between bacterial phyla and carotenoid structures.

## 1. Introduction

Some species of bacteria, yeast, and fungi, as well as algae and higher plants, synthesize a large number of carotenoids with different molecular structures, and more than 750 carotenoids with different structures have been isolated from natural sources [1]. Many beneficial pharmaceutical effects of carotenoids have recently been reported*.* Therefore, evaluating the pharmaceutical potentials of various carotenoids may represent an interesting field in medical research. However, the number of carotenoid species that have been examined for this purpose has been limited, and has included C_40_ carotenoids possessing skeletons composed of 40 carbon atoms, such as dicyclic carotenoids, e.g., β-carotene, α-carotene, β-cryptoxanthin, zeaxanthin, lutein, canthaxanthin, astaxanthin, and fucoxanthin, and the acyclic carotenoid lycopene [2,3,4,5,6,7,8]. Difficulties have been associated with identifying natural sources to supply sufficient amounts of new or rare carotenoids, with the exception of carotenoids that can be isolated from a species of higher plants or algae or chemically synthesized. It has therefore been desirable to find cultivable bacteria that produce new or rare carotenoids, since they can easily be reproduced. 

Marine bacteria have not been examined as extensively as land bacteria. Thus, the Marine Biotechnology Institute Co., Ltd. (MBI, Kamaishi, Japan) was established in December, 1988, and continued to isolate novel or rare marine bacteria until March, 2008, the number of which reached more than ten thousand [9,10,11,12]. Many bacteria have been shown to produce dicyclic or monocyclic C_40_ carotenoids, in addition to some acyclic C_30_ carotenoids with a 30 carbon skeleton [1,13]. The MBI isolated new or rare dicyclic C_40_ carotenoids with the β-carotene (β,β-carotene) skeleton from Gram-negative marine bacteria belonging to the class α-Proteobacteria, phylum Proteobacteria, e.g., astaxanthin glucoside from *Paracoccus* sp. strain N81106 (re-classified from *Agrobacterium aurantiacum*) [14], 2-hydroxyastaxanthin from *Brevundimonas* sp. strain SD212 [15], and 4-ketonostoxanthin 3′-sulfate from *Erythrobacter* sp. strain. PC6 (re-classified from *Flavobacterium* sp. PC-6; MBIC02351) [16]. These marine bacteria were also able to produce astaxanthin [17]. The carotenoid biosynthesis gene clusters of these marine bacteria have been elucidated in detail [17,18,19]. 

The generation of free radicals has been suggested to play a major role in the progression of a wide range of pathological disturbances, including myocardial and cerebral ischemia [20], atherosclerosis [21], renal failure [22], inflammation [23], and rheumatoid arthritis [24]. The subsequent peroxidative disintegration of cells and organelle membranes has also been implicated in various pathological processes [25]. Carotenoid pigments, which have been shown to possess strong antioxidant activities, have been attracting increasing attention due to their beneficial effects on human health, e.g., their potential to prevent diseases such as cancer and cardiovascular diseases [26]. 

We have attempted to identify novel or rare types of carotenoids from yellow or red pigment-producing marine bacteria that were classified to belong to rare or novel species by 16S rRNA analyses since 2002. The results of this screening led to the isolation of diapolycopenedioc acids xylosylesters A–C (new carotenoids) from *Rubritalea squalenifaciens* [27,28], methyl 5-glucosyl-5,6-dihydro-apo-4,4′-lycopenoate (a new carotenoid) from *Planococcus maritimus* [29], and (3*R*)-saproxanthin and (3*R*,2′*S*)-myxol (rare carotenoids) from a novel species belonging to the family Flavobacteriaceae [30]. In this review, we report the structures and antioxidant activities of these carotenoids, and consider relationships between bacterial phyla and carotenoid structures. 

## 2. Results

### 2.1. Diapolycopenedioc Acid Xylosylesters A–C from Rubritalea Squalenifaciens [27,28]

A yellow pigment-producing bacterium (strain HOact23^T^) that was found to produce squalene was isolated from the homogenate of the marine sponge *Halicondria okadai*, which had been collected from the Miura peninsula (Kanagawa, Japan), and was subsequently classified as a novel species in the genus *Rubritalea*, belonging to phylum Verrucomicrobia, based on 16S rRNA gene sequence data. The name proposed for the new taxon was *Rubritalea squalenifaciens* [31], with the type strain HOact23^T^ (=MBIC08254^T^ = DSM 18772^T^). 

**Figure 1:**
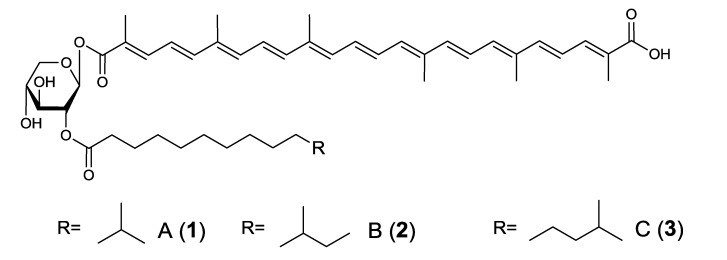
The structures of diapolycopenedioc acids A (**1**), B (**2**) and C (**3**).

*R. squalenifaciens* was cultured in 100 mL of medium (1.0% starch, 0.4% yeast extract, and 0.2% peptone in seawater) in a 500 mL Erlenmeyer flask at 30 °C on a rotary shaker at 120 rpm for 2 days, and the carotenoids produced were purified from the cells using chromatographic methods (EtOAc/H_2_O partition → silica gel column chromatography CH_2_Cl_2_–MeOH (20:1) → preparative silica gel HPLC CH_2_Cl_2_–MeOH (15:1) → preparative ODS HPLC (MeOH)). Three carotenoids were purified from cells in the 42-liter culture (diapolycopenedioc acids xylosylesters A (**1**) 10.2 mg, B (**2**) 3.0 mg, and C (**3**) 2.2 mg, respectively). The structures of compounds **1**–**3** were determined by HRESI-MS and spectroscopic (UV-Vis, NMR (1D and 2D investigations on ^1^H and ^13^C nuclei), and [α]_D_) analyses as shown in Figure 1. Compounds **1**–**3** were all new carotenoids. 

Compounds **1**–**3** possessed diapolycopenedioc acid (C_30_ carotenoid) [32,33] as their aglycone. Diapolycopenedioic acid glucosyl ester and diapolycopenedioic acid diglucosyl were previously shown to be carotenoids that possessed diapolycopenedioc acid as the aglycone [32]. Compounds **1**–**3** were the first carotenoids to include 2-acyl-d-xylose in their structures. 

The antioxidant activity of compound **1** was evaluated using ^1^O_2_ suppression activity. Its IC_50_ was 5.1 μM (the IC_50_ values of astaxanthin and β-carotene were 8.9 μM and >100 μM, respectively). 

### 2.2. Methyl 5-Glucosyl-5,6-Dihydro-Apo-4,4′-Lycopenoate from Planococcus Maritimus [29]

A yellow pigment-producing bacterium (strain iso-3), which was found to be solvent-tolerant, was isolated as an orange-pigmented colony from the microbial analysis of a sample derived from an intertidal sediment from the Clyde estuary, UK. The 16S rRNA gene sequence of strain iso-3 was the most similar to that of type strain *Planococcus maritimus* (99.5 as a similarity score, and 96.4 as an s_ab score, from the Sequence match analysis of RDP), which belongs to the class Bacilli, phylum Firmicutes, and was identified as *Planococcus maritimus* strain iso-3.

**Figure 2:**
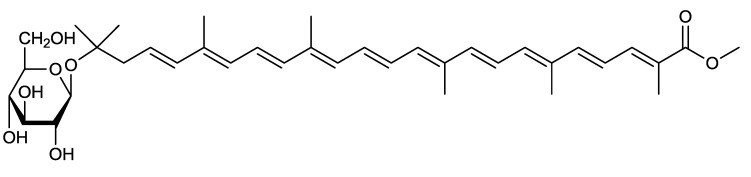
The structure of methyl 5-glucosyl-5,6-dihydro-apo-4,4′-lycopenoate (**4**).

Strain iso-3 was cultured in 100 mL of medium (Marine Broth 2216, Difco, Sparks, MD, USA) in a 500 mL Erlenmeyer flask at 30 °C on a rotary shaker at 120 rpm for 1 day, and the carotenoid produced was purified from the alkaline-digested cells using chromatographic methods (EtOAc/H_2_O partition → silica gel column chromatography (CH_2_Cl_2_–MeOH (10:1) → preparative silica gel HPLC CH_2_Cl_2_–MeOH (10:1) → preparative ODS HPLC (96% MeOH)). A total of 2.5 mg of pure methyl 5-glucosyl-5,6-dihydro-apo-4,4′-lycopenoate (**4**) was obtained from the cells in the 18-liter culture, and the structure of compound **4** was determined by HRESI-MS and spectroscopic (UV-Vis, NMR (1D and 2D investigations on ^1^H and ^13^C nuclei), and [α]_D_) analyses, as shown in Figure 2. Compound **4** was a new carotenoid. Compound **4** possessed 5,6-dihydro-5-hydroxy-apo-4, 4′-lycopene-4′-oic acid (C_30_ carotenoid) as its aglycone. Although 4,4′-diapocarotene-4-oic acid [32] was previously reported to be a related C_30_ carotenoid aglycone, 5,6-dihydro and 5-hydroxy functions in the aglycone of compound **4** were demonstrated for the first time. The antioxidant activity of compound **4** was evaluated using ^1^O_2_ suppression activity, and its IC_50_ value was 5.1 μM. We previously described the isolated carotenoid as methyl glucosyl-3,4-dihydro-apo-8′-lycopenoate [29], but confirmed its structure as methyl 5-glucosyl-5,6-dihydro-apo-4,4′-lycopenoate, as shown in this review. Corrigenda is currently being prepared for the previous study. 

### 2.3. (3R)-Saproxanthin and (3R,2′S)-Myxol [30]

Strain 04OKA-13-27 (MBIC08261) was isolated from the dense mats of filamentous algae from within the territory of damselfish (*Stegastes nigricans*). Strain YM6-073 (MBIC06409) was isolated from a sediment sample collected 0.1 m below the surface of the sea by cultivating for 30 days on an HSV medium. The two marine bacteria, which had been collected off the coast of Okinawa Prefecture, were classified on the basis of this 16S rRNA gene sequences. A similarity search in the databases of the DNA Data Bank of Japan (DDBJ) and RNA Database Project II (RDPII) showed the 16S rRNA gene sequences of the both strains (04OKA-13-27 and YM6-073) to be 96.5% (1408 bp/1459 bp) similar to *Stanierella latercula* ATCC 23177^T^, 95.5% (1324 bp/1386 bp) similar to *Gaetbulimicrobium* *brevivitae* strain SMK-19^T^, and 94.2% (1306 bp/1386 bp) similar to *Robiginitalea biformata* strain HTCC2501^T^. The phylogenetic relationship between these strains was deduced with already known species in the family Flavobacteriaceae. The result obtained revealed that the two bacterial strains should be classified as novel species of the family Flavobacteriaceae. 

**Figure 3:**
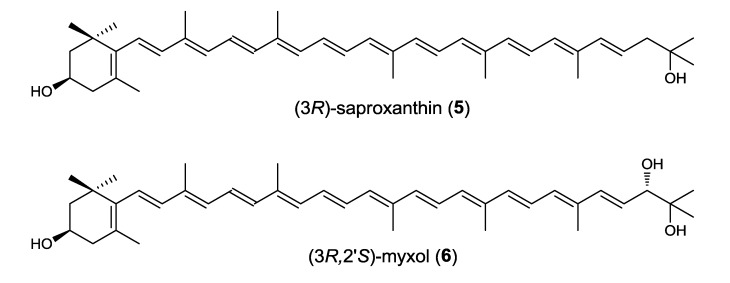
The structures of (3*R*)-saproxanthin (**5**) and (3*R*,2′*S*)-myxol (**6**).

Both 04OKA-13-27 and YM6-073 were cultured in 100 mL of medium (Marine Broth 2216, Difco) in a 500 mL Sakaguchi flask at 30 °C on a rotary shaker at 100 rpm for 1 day, and the carotenoids produced were each purified from the cells using chromatographic methods (EtOAc/H_2_O partition → silica gel column chromatography hexane–ethyl acetate (2:1) → preparative silica gel high performance thin layer chromatography (HPTLC; Merck, Darmstadt, Germany) CH_2_Cl_2_–MeOH (10:1) → preparative ODS HPLC (MeOH)). A total of 0.3 mg (04OKA-13-27) and 0.5 mg (YM6-073) of pure carotenoids were obtained from the cells of each 2 liter culture, and the carotenoids were identified as (3*R*)-saproxanthin (04OKA-13-27) (**5**) and (3*R*,2′*S*)-myxol (YM6-073) (**6**) by MS, ^1^H-NMR, and CD analyses, respectively (Figure 3). 

The antioxidative activities of compounds **5** and **6** were examined using rat brain homogenate model. Compounds **5** and **6** showed potent antioxidant activities (IC_50_ 2.1 μM (**5**) and 6.2 μM (**6**)) (IC_50_ 10.9 μM (β-carotene)). 

## 3. Discussion

The MBI has isolated approximately 1000 pigment-producing marine bacteria. We selected 10 strains, which were identified as rare or novel species by 16S rRNA, including strain HOact23^T^ (*Rubritalea squalenifaciens* sp. nov., phylum Verrucomicrobia), strain iso-3 (*Planococcus maritimus*, the class Bacilli, phylum Firmicutes), strain 04OKA-13-27 (novel species of the family Flavobacteriaceae), and strain YM6-073 (novel specie of the family Flavobacteriaceae) from these isolated bacteria.

We found two-type new C_30_ carotenoids diapolyconedioc acid xylosylesters (compound **1**–**3**) from HOact23^T^ and methyl 5-glucosyl-5,6-dihydro-apo-4,4′-lycopenoate (compound **4**) from iso-3 through the isolation and structural analyses of carotenoids produced by these strains. Acyclic C_30_ carotenoids were previously shown to be contained in land bacteria including *Staphylococcus aureus*, belonging to the class Bacilli, and the methanotrophs *Methylobacterium rhodium* (formerly *Pseudomonas rhodos*), belonging to the class α*-*Proteobacteria, and *Methylomonas* sp., belonging to the class γ-Proteobacteria [17,34]. Thus, acyclic C_30_ carotenoids are likely to widely exist in domain bacteria (prokaryotes), *i*.*e*., they are present not only in some low-GC Gram-positive bacteria, but also in some phyla in Gram-negative bacteria. The strong singlet-oxygen-quenching activities of our C_30_ carotenoids also indicated that such C_30_ carotenoids are promising as functional carotenoids, although these *in vivo* functional analyses have not yet been conducted. 

We isolated two rare monocyclic C_40_ carotenoids with one 3-hydroxy-β-ring ((3*R*)-saproxanthin (compound **5**) from 04OKA-13-27 and (3*R*,2′*S*)-myxol (compound **6**) from YM6-073), which belong to the family Flavobacteriaceae, phylum Bacteroidetes. (3*R*)-Saproxanthin has only previously been detected from *Saprospira grandis*, which belongs to the family Saprospiracea, phylumBacteroidetes [35]. Hence, marine bacterial strain 04OKA-13-27 was the second species to produce saproxanthin. (3*R*,2′*S*)-Myxol has only previously been detected in marine bacterial strain P99-3 (MBIC03313), belonging to the family Flavobacteriaceae [15], and in the cyanobacterium *Anabaena variabilis* ATCC 29413, phylum Cyanobacteria [36]. Therefore, marine bacterial strain YM6-073 was the third species to produce myxol. Myxoxanthophyll (myxol 2′-fucoside), which is widely distributed in phylum Cyanobacteria, contains myxol as its aglycone. These findings indicated that such monocyclic C_40_ carotenoids with one 3-hydroxy-β-ring exist in phylum Bacteroidetes as well as phylum Cyanobacteria. 

The carotenoids produced by the six other strains isolated were all zeaxanthin, which is a common carotenoid in domain bacteria. Our study may be effective for identifying rare and new carotenoids based on its ratio (4/10). In addition, all the rare and new carotenoids (**1**–**6**) isolated possessed potent antioxidant activities. 

## 4. Conclusions

Marine bacteria are likely to produce carotenoids to protect themselves from activated oxygen produced by sunlight (mainly ^1^O_2_); therefore, their potent antioxidant activities were expected and reasonable. Therefore, the techniques performed in our study effectively identified new antioxidant carotenoids.
